# Multiorgan failure due to hemophagocytic syndrome: A case report

**DOI:** 10.1186/1757-1626-1-209

**Published:** 2008-10-03

**Authors:** Juan Mayordomo-Colunga, Corsino Rey, Soledad González, Andrés Concha

**Affiliations:** 1 Paediatric Intensive Care Unit, Department of Paediatrics, Hospital Universitario Central de Asturias, University of Oviedo, Oviedo, Spain; 2 Department of Hematology, Hospital Universitario Central de Asturias, University of Oviedo, Oviedo, Spain

## Abstract

**Introduction:**

Hemophagocytic syndrome (HFS) is a potentially lethal disorder due to an uncontrolled immune response to a triggering agent. Our objective is to raise the importance of HFS early diagnosis by presenting a representative case.

**Case presentation:**

A sixteen-year-old girl with Still disease diagnosis developed a progressive multiorgan failure including acute respiratory distress (ARDS), anemia and thrombopenia, elevated liver enzymes, renal failure, coagulopathy with hypofibrinogenemia, and acute phase reactants elevation despite broad-spectrum antibiotics. A bone marrow puncture-biopsy was performed, and hemophagocytosis was found. Prolonged fever, splenomegaly, bicytopenia, hypofibrinogenemia, hyperferritinemia and hypertriglyceridemia confirmed HFS diagnosis. She received intensive care support therapy including mechanical ventilation and specific therapy according to HLH 2004 protocol, with a very good response.

**Conclusion:**

Our case shows complexity of HFS diagnosis, due to septic shock-like manifestations. Early diagnosis is essential to start appropriate treatment achieving a better outcome.

## Introduction

Hemophagocytic syndrome (HFS) is a rare disorder characterized by prolonged fever, cytopenias, hepatosplenomegaly, hypertriglyceridemia, disseminated intravascular coagulation (DIC)-like coagulopathy and bone marrow, spleen, liver or lymphatic nodes histiocytosis [1-3]. A sudden presentation, like a septic shock is possible making its early recognition a challenging diagnosis [4]. It is well known that HFS could be a severe complication in some infections (mainly virals) or in some underlying diseases, such as chronic juvenile arthritis (CJA) [3,5,6]. Moreover, it is one of the differential diagnosis in fever of unknown origin [7].

Taking into account that a better outcome has been related to an early treatment [3,8], presentation of a difficult diagnosis case in a young lady could be helpful and interesting.

## Case presentation

A sixteen-year-old girl presented with rash, elevated fever and joints swelling. She was admitted to the hospital to make further examinations as the symptoms persisted for 15 days. A diagnosis of probable Still disease was made. In the following days, her clinical state worsened, with persistence of elevated fever. Serologic tests were negative for *Bartonella*, *Parvovirus *B19, *Rickettsia*, viral hepatitis (A, B and C), HIV, *Toxoplasma*, *Salmonella *and *Yersinia*. Blood cultures were also negative. No abnormalities were found in abdominal and cardiac ultrasonography and in cranial computed tomography (CT). A bone gammagraphy, showed enhanced captation in right tibial malleolus and in proximal interphalangeal joints of fingers 2 and 4 of the right hand, and finger 4 of the left hand. An abdominal CT showed a biliary bladder wall enlargement and a laparotomy was performed, but no signs of cholecystitis were found. A broad spectrum antibiotherapy was started with ciprofloxacin and imipenem. The patient was transferred to pediatric intensive care unit (PICU) as a consequence of progressive multiorgan failure including acute respiratory distress syndrome, liver failure, anemia, thrombopenia and increasing acute phase reactants (APR). On admission to PICU, the patient was on mechanical ventilation. Examination revealed generalized oedema, hypoventilation in both lung bases, abdominal distension and hepatoesplenomegaly. There was also active bleeding around puncture points and through nasogastric tube. Blood analysis on admission is shown in table 1.

**Table 1:** Blood analysis on admission

Hemoglobin	7,7 g/dL
Hematocrit	22,7%
Leucocytes	12.200/mm^3 ^(with a marked left shift)
Platelets	29.000/mm^3^
Prothrombin rate	75%
Fibrinogen	85 mg/dL
D-dimers	6.593 ng/mL
Aspartate aminotransferase	3.257 U/L
Alanine aminotransferase	907 U/L
Direct bilirrubin directa	9,2 mg/dL
Ammonium	57 μmol/L
Creatine kinase	1.067 U/L
Dehydrogenase lactate	19.747 U/L
Lipase	48 U/L
Amylase	137 U/L
Sodium	144 mEq/L
Potassium	3,1 mEq/L
Urea	63 mg/dL
Creatinine	1,1 mg/dL
Procalcitonin	3,03 ng/mL
C reactive protein	16,7 mg/dL

A high positive end-expiratory pressure (PEEP) was set due to hypoxemia (up to 14 cmH_2_O), with a PO_2_/FiO_2 _of 141. Thorax radiography showed bilateral diffuse infiltrates, and slight cardiomegaly. Inotropic support was needed (dopamine at 15 μg/kg/minute) and a perfusion with furosemide (0.5 mg/kg/hour) was started. Laboratory analysis showed abnormal values for haemoglobin (7.7 g/dL), platelets (29,000/mm^3^) and coagulation times including hypofibrinogenemia (85 mg/dL). She received red blood cell concentrates, platelets and fresh frozen plasma. The same antibiotherapy was maintained and acute liver failure support treatment was started.

**Figure 1:**
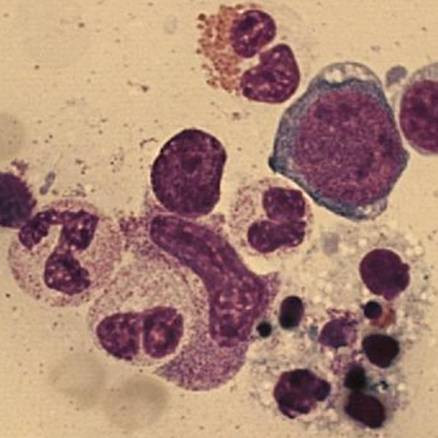
**Bone marrow smear (optic microscope)**. Activated macrophages and hemophagocytosis from bone marrow puncture-biopsy.

A bone marrow puncture-biopsy was performed. Activated macrophages with hemophagocytosis were found (figure 1), which together with the clinical and analytical data (bicytopenia, and coagulopathy with hypofibrinogenemia, and afterwards a ferritin of 190594 μg/L and triglycerides of 677 mg/dL) confirmed the HFS diagnosis. Specific treatment for this syndrome was started, following Hemophagocytic Lymphohistiocytosis (HLH) 2004 guidelines. She had a very good response, and at forth day from admission she was extubated to non invasive ventilation using a full-face mask. A progressive analytical normalization was observed and she was discharged after 12 days in PICU, without any sequelae.

She is currently being followed as an outpatient. She had two reactivations of her rheumatoid disorder, with a good response to corticoids.

## Discussion

HFS is an activation of mononuclear phagocyte system cells, with hemophagocytosis in bone marrow and the rest of reticuloendothelial system. This syndrome can be either primary/familial (familial hemophagocytic lymphohistiocytosis – FHL) or reactive/secondary. FHL has a recessive autosomal inheritance and it develops in children younger than 2 years, even though in rare cases it can feature later on [9]. It is rapidly lethal and it is sometimes related to some immunological diseases (X-linked lympho-proliferative, Chediak Higashi and Griscelli syndromes).

Secondary HFS has a better outcome than primary HFS. It is triggered mainly by viral infections (especially Ebstein-Barr virus) [10], and also by bacterial, parasitic and fungal infections. It can also develop during malignancies and rheumatoid disorders (kwown in this case as macrophagic activation syndrome), as in our patient [6].

The activation of mononuclear system cells occurs due to a hypersecretion of proinflammatory cytokines (IFNγ, TNFα, IL6, IL10, M-CSF), as a consequence of a triggering agent, which is often a viral infection [11]. The underlying problem is a T and Natural Killers cells dysfunction, which leads to an uncontrolled immunological response [12] due to inability to eliminate the triggering agent. All viral, bacterial, parasitic and fungal cultures performed in our case were negative.

Impaired perforine function due to gene mutations seems to play an important role in HFS pathogenesis, as reported in literature [12]. They are implicated in cytotoxicity by forming a death-inducing pore in target cell [13].

HFS diagnosis is made basing on clinical and histological criteria. Five out of 8 criteria must be fulfilled. Absence of hemophagocytosis does not exclude the diagnosis [2]. Multiorgan failure is the most severe presentation of HFS. In pediatrics, multiorgan failure is usually caused by sepsis. In the present case, the initial diagnosis was septic shock. Therefore, HFS has to be included between the causes of multiorgan failure in pediatrics to permit an early diagnosis and treatment. Central nervous system (CNS) is often involved, which has been linked with a poor prognosis [14]. Even though our patient developed a very severe form of HFS, there seemed to be no CNS involvement, and this agrees with the good outcome.

Treatment is nowadays applied according to HLH 2004 protocol, which is designed for primary HFS and also used in severe secondary HFS cases. Aggressive immunochemotherapy is given (dexamethasone, cyclosporine A, etoposide and in patients with CNS symptoms or abnormal CSF, also intrathecal methotrexate and corticoids) [2]. After initial treatment, bone marrow transplantation is indicated in primary disease and in severe and persistent secondary HFS [1].

Without treatment, this is a lethal disorder. Mortality is high depending in reactive HFS on underlying disorder. In Henter's study, in which 113 patients under 15 years of age were included, survival rate at 3.1 years was 51% in primary cases, while it was 55% in reactive ones. All patients had received treatment according to HLH 1994 protocol [1].

## Conclusion

Our patient had a very good outcome, without any sequelae. Early recognition of this syndrome to apply specific therapy as well as multiorganic failure treatment in PICU, are management key factors. HFS is probably underdiagnosed, as multiorgan failure is usually explained by other more common causes like septic shock. [4]

## Abbreviations

APR: acute phase reactants; CJA: chronic juvenile arthritisl CNS: central nervous system; CSF: cerebrospinal fluid; CT: computed tomography; DIC: disseminated intravascular coagulation; FHL: familial hemophagocytic lymphohistiocytosis; HFS: hemophagocytic syndrome; HLH: Hemophagocytic Lymphohistiocytosis; IFN: interferon; IL: interleukin; M-CSF: macrophage colony-stimulating factor; PEEP: positive end-expiratory pressure; PICU: pediatric intensive care unit; PO_2_/FiO_2_: partial pressure of arterial oxygen/fraction of inspired oxygen ratio; TNF: tumor necrosis factor.

## Competing interests

The authors declare that they have no competing interests.

## Authors' contributions

JM-C, CR, SG and AC were responsible for the diagnosis and treatment of the described patient. JM-C, CR and SG performed the literature research and drafted the manuscript, which was read and approved by all authors in its final version.

## Consent

Written informed consent was obtained from the patient and his parents for publication of this case report and any accompanying images. A copy of the written consent is available for review by the Editor-in-Chief of this journal.

## References

[B1] Henter JI, Samuelsson-Horne A, Arico M, Egeler RM, Elinder G, Filipovich AH, Gadner H, Imashuku S, Komp D, Ladisch S, Webb D, Janka G (2002). Treatment of hemophagocytic lymphohistiocytosis with HLH-94 immunochemotherapy and bone marrow transplantation. Blood.

[B2] Henter JI, Horne A, Arico M, Egeler RM, Filipovich AH, Imashuku S, Ladisch S, McClain K, Webb D, Winiarski J, Janka G (2007). HLH-2004: Diagnostic and therapeutic guidelines for hemophagocytic lymphohistiocytosis. Pediatr Blood Cancer.

[B3] Cortis E, Insalaco A (2006). Macrophage activation syndrome in juvenile idiopathic arthritis. Acta Paediatr.

[B4] Stephan JL, Galambrun C (2000). Syndrome d'activation lymphohistiocytaire chez 1'enfant. Arch Pediatr.

[B5] Stephan JL, Kone-Paut I, Galambrun C, Mouy R, Bader-Meunier B, Prieur AM (2001). Reactive haemophagocytic syndrome in children with inflammatory disorders. A retrospective study of 24 patients. Rheumatology (Oxford).

[B6] Stabile A, Bertoni B, Ansuini V, La Torraca I, Salli A, Rigante D (2006). The clinical spectrum and treatment options of macrophage activation syndrome in the pediatric age. Eur Rev Med Pharmacol Sci.

[B7] Fadilah SA, Raymond AA, Leong CF, Cheong SK (2006). Haemophagocytic syndrome presenting as pyrexia of unknown origin. Med J Malaysia.

[B8] Ariffin H, Lum SH, Cheok SA, Shekhar K, Ariffin WA, Chan LL, Lin HP (2005). Haemophagocytic lymphohistiocytosis in Malaysian children. J Paediatr Child Health.

[B9] Allen M, De FC, Legrand F, Clementi R, Conter V, Danesino C, Janka G, Arico M (2001). Familial hemophagocytic lymphohistiocytosis: how late can the onset be?. Haematologica.

[B10] Janka GE (2007). Hemophagocytic syndromes. Blood Rev.

[B11] Janka GE (2007). Familial and acquired hemophagocytic lymphohistiocytosis. Eur J Pediatr.

[B12] Goransdotter EK, Fadeel B, Nilsson-Ardnor S, Soderhall C, Samuelsson A, Janka G, Schneider M, Gurgey A, Yalman N, Revesz T, Egeler R, Jahnukainen K, Storm-Mathiesen I, Haraldsson A, Poole J, de Saint BG, Nordenskjold M, Henter J (2001). Spectrum of perforin gene mutations in familial hemophagocytic lymphohistiocytosis. Am J Hum Genet.

[B13] Lowin B, Peitsch MC, Tschopp J (1995). Perforin and granzymes: crucial effector molecules in cytolytic T lymphocyte and natural killer cell-mediated cytotoxicity. Curr Top Microbiol Immunol.

[B14] Hallahan AR, Carpenter PA, O'Gorman-Hughes DW, Vowels MR, Marshall GM (1999). Haemophagocytic lymphohistiocytosis in children. J Paediatr Child Health.

